# Behavioral Profiling in Zebrafish Identifies Insecticide-Related Compounds

**DOI:** 10.1021/acs.jafc.4c09342

**Published:** 2025-01-24

**Authors:** Gerald Watson, Jack Taylor, William T Lambert, Kenneth Beavers, Daniel Kirk, Martin J Walsh, David Kokel, Matthew N McCarroll

**Affiliations:** 1 Corteva Agriscience, 9330 Zionsville Road, Indianapolis, Indiana 46268, United States; 2 Institute for Neurodegenerative Diseases, University of California, San Francisco, San Francisco, California 94158, United States; 3 Department of Pharmaceutical Chemistry, University of California, San Francisco, San Francisco, California 94158, United States

**Keywords:** zebrafish, behavioral profiling, endosulfan, high throughput screening, pesticide discovery, pest management, GABA

## Abstract

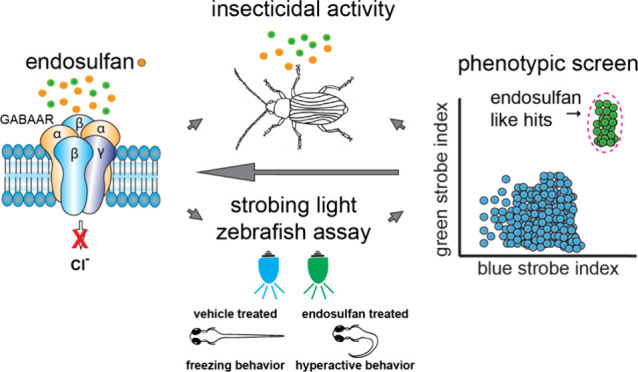

Pesticides, including insecticides, are indispensable for large-scale agriculture. Modulating chloride ion channels has proven highly successful as a mode of action (MoA) for insect management. Identifying new ligands for these channels affords opportunities for the potential development of new insecticide products. We discovered an unexpected behavioral response in larval zebrafish exposed to endosulfan, a γ-aminobutyric acid (GABA)-gated chloride channel modulator. At low concentrations, endosulfan increased zebrafish motor activity under strobing blue or green light stimuli. Using this distinct behavioral phenotype as a proxy for similar modes of action, we screened over 9,000 compounds and identified several structurally diverse hits that phenocopied endosulfan’s effects *in vivo*. Nine selected hits were tested in an *in vitro* GABA receptor (GABAR) oocyte assay, confirming that certain compounds block Drosophila GABAR activation. Subsequent *in vivo* insect assays revealed that one of these GABAR antagonists showed insecticidal activity against the Western corn rootworm (*Diabrotica virgifera virgifera*), which is a commercially important pest of corn. Our findings demonstrate a new approach for identifying GABAR-targeting insecticides through behavior-based screening in zebrafish.

## Introduction

The challenge of developing new, safe, and sustainable insect control agents to protect plants from insect damage is formidable and costly.^1,2^ A traditional approach to the development of a new insect control agent is to first identify a novel chemical moiety and then, through synthetic iterations, build in the potency, spectrum, selectivity, and safety required of modern crop protection products. The methods of discovery of these novel synthetic starting points vary in the details, but typically involve the identification of weak insecticidal activity through screening of large numbers of chemical candidates, either directly against target insect pests, or against convenient surrogate insects.^3−5^ This approach has proven successful with the discovery of many, if not most, important insecticides over the last several decades.^6,7^ Nonetheless, the development of new approaches for insecticidal hit identification is an important goal that can yield unique structures from which to develop novel insect control agents.

In the development of new pharmaceutical agents, many of the technical challenges and high costs of *in vivo* studies in neuropharmacology can be circumvented using zebrafish, a simple vertebrate alternative to rodents.^8^ Zebrafish are typically used as a model organism to assess vertebrate risk and environmental toxicology.^9−11^ To expand and accelerate neuropharmacological studies, a platform has been built in which the behavior of zebrafish under the influence of neuroactive small molecules can be monitored and measured systematically.^12^ Behavioral profiling in zebrafish can be used for high-throughput screening and/or as the basis for formulating hypotheses that subsequently can be tested using appropriate *in vitro* systems. This approach has been successful in identifying new small molecules in the class of antipsychotics, sedatives and anesthetics.^12,13^ In addition, these approaches have been used to help characterize antiaddictive compounds.^14^ Using custom built hardware, we have created a unique battery of assays that expose the animals to light or vibrational stimulus at different times.^12,15^ From recorded video sequences, the animal’s behavioral profile can be constructed using a motion index metric.^12^ Interestingly, neuroactive compounds can alter the behavioral response of zebrafish larvae, and often drugs operating by the same mechanism of action (MoA) will cluster together when comparing the constructed behavioral profiles.^12,16,17^ This information can then be utilized to discover new compounds with similar utility and/or MoA.

Neuroactive compounds that interact with the γ-aminobutyric acid (GABA) system as inverse agonists have been previously reported to have strong phenotypes in the zebrafish (i.e., pentylenetetrazol (PTZ), and picrotoxin (PTX)).^18,19^ Chloride channels as a class represent attractive biological targets for drug development.^20^ These channels mediate a wide variety of physiological functions and are conserved throughout evolution. Examples of small molecule function on chloride channels have been exemplified in behavioral experiments with zebrafish larvae. Inverse agonists such as PTZ and PTX cause hyperexcitability in both zebrafish and rodent models and are capable of inducing seizure-like behaviors.^21^ PTZ and PTX also induce large increases in neuronal activity of zebrafish larvae as seen in live calcium imaging and antibody labeling of the neuronal activity marker pERK.^14,22−25^ Recently, behavioral drug-screening assays have been used in zebrafish to identify novel anesthetics, anxiolytics, hypnotics and analgesics.^12,26^ Many of these identified compounds appear to be acting on chloride channels, indicating zebrafish larvae as promising organisms in which to discover novel chloride channel modulators.

**Figure 1:**
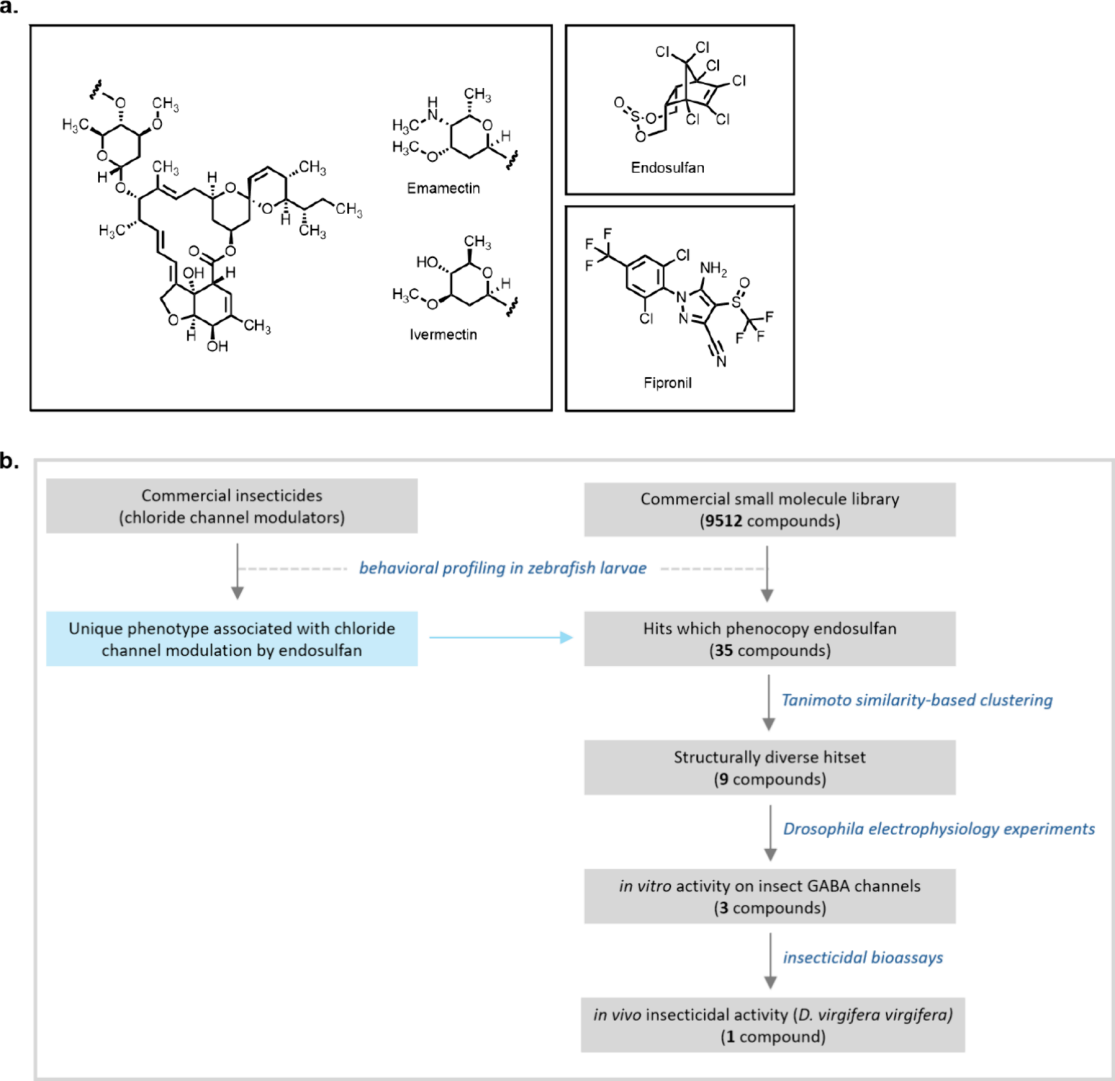
Overview of a zebrafish behavioral profiling study of insecticides. (a) Structures of insecticides acting on chloride channels. (b) Workflow for the present study.

Chloride channels have long been exploited as insecticidal target sites, as well.^27^ In fact, insecticides acting at both glutamate receptors (GluRs) and γ-aminobutyric acid receptors (GABAR) are still widely used today, as demonstrated by emamectin, ivermectin, endosulfan, and fipronil (Figure. 1a). We hypothesized that these compounds could be used as starting points for the identification of novel insecticide hits by using their zebrafish behavioral phenotypes as a screening tool to select candidate molecules for further testing. In the present study, the zebrafish phenotypes induced by these insecticides were characterized. It was found that the GABAR antagonist endosulfan produced a strong and unique phenotype relative to the other chloride channel effectors. Next, we searched our high-throughput screening database to identify compounds from a previously screened commercially available small molecule library that produce phenotypes similar to endosulfan in zebrafish. From this search, nine compounds with structural diversity from both endosulfan and each other were selected for evaluation in oocyte-based *Drosophila melangaster* GABAR (RDL) electrophysiology experiments. These studies identified three compounds that block insect GABAR activation. This subset was then assessed in whole organism insecticide screens, resulting in the discovery of a new chemical starting point for the control of Western corn rootworm (*Diabrotica virgifera virgifera*), which is a commercially important insect pest. Figure 1b outlines the present study in flowchart form.

## Materials and Methods

### Fish Maintenance, Breeding, and Chemical Treatments

Maintenance and breeding of wild type Singapore zebrafish were performed as described^28^ and staged in days post fertilization (dpf). All embryos were raised on a 14/10-h light/dark cycle at 28 °C until 7 dpf. Larvae were distributed 8 animals per well into square 96-well plates (GE Healthcare Life Sciences) with 300 μL of egg water. Chemical treatments were applied directly to the egg water and larvae were incubated at room temp for 1 h before behavioral analysis.

### Chemical Treatments and Libraries

Technical samples of emamectin, ivermectin, endosulfan, and fipronil were purchased directly from ChemServices. These reference compounds were dissolved in dimethyl sulfoxide (DMSO) at a stock concentration of 30 mM and assayed in 3–12 replicate well plates in a dose response with concentrations ranging from 195.31 nM - 600 μM.

The Chembridge library (DiverSet, Chembridge Corporation) contains 10,000 compounds at 10 mM in DMSO. All compounds were diluted in egg water and screened at 30 μM final concentration in <1% DMSO. Controls were treated with an equal volume of DMSO. All Chembridge library compounds were ordered from the Chembridge online market (www.hit2lead.com) and validated in 3–12 replicate well plates in a dose response behavioral assay with concentrations ranging from 1.56 μM-100 μM.

### Automated Behavioral Phenotyping Assays

Plates were illuminated with a 760 nm infrared light and digital video was captured at 25 frames per second using an AVT Pike digital camera (Allied Vision). Behavioral assays were run in tandem to create a full behavioral battery. Duration of individual assays was 30–120 s consisting of a combination of acoustic and light stimuli, strobing light stimuli were delivered in blue (560 nm, 18 μW/mm^2^) and in green (525 nm, 11 μW/mm^2^) at 4 Hz for 120 s for each assay. For more details on custom hardware and assay design see McCarroll et al., 2019.^12^ Zebrafish motion index (MI) was quantified by frame differencing and then normalized to calculate the resulting MI for individual wells as follows: MI = sum(abs(frame_n_ – frame_n–1_)) or in some instances as cd(10).^15^ Detailed descriptions of the analysis are described here.^12,13,15,29^

### Phenotypic Metrics

To identify screening compounds that elicited strobe-related behaviors, a score was calculated for the blue and green strobe response (strobe score) by averaging the maximum motion index value during 6, 5 s instances across the strobe assay duration. Values were then normalized from 0 to 1 using the scikit function *sklearn.preprocessing.normalize* written for python. Structural clustering was performed on the 9 hit compounds we followed up on using the rdkit function *FingerprintMols* package written for python. Tanimoto similarity function was used with a threshold of 0.45 to define clusters and visualized using the SciPy hierarchy dendrogram function. T-distributed Stochastic Neighbor Embedding (t-SNE) and random forest analysis were performed using the scikit learn (sklearn) library functions in python using raw MI data for the respective treatments.

### Data Visualization

**Figure 2:**
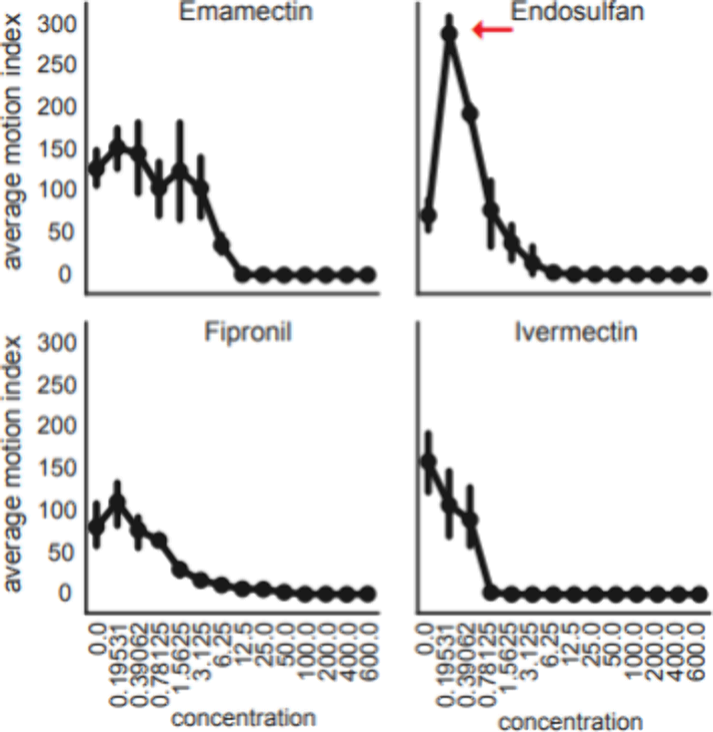
Dose response of insecticides targeting chloride channels in zebrafish larvae. Line plot of average motion index (*y*-axis) showing zebrafish larvae motor activity at increasing concentrations (*x*-axis) of 4 insecticides. Red arrow indicates significantly increased motor activity in low concentration endosulfan treated animals. Error bars denote 95% confidence interval around the estimated mean (*n* = 12 wells/condition and 8 larvae/well).

**Figure 3:**
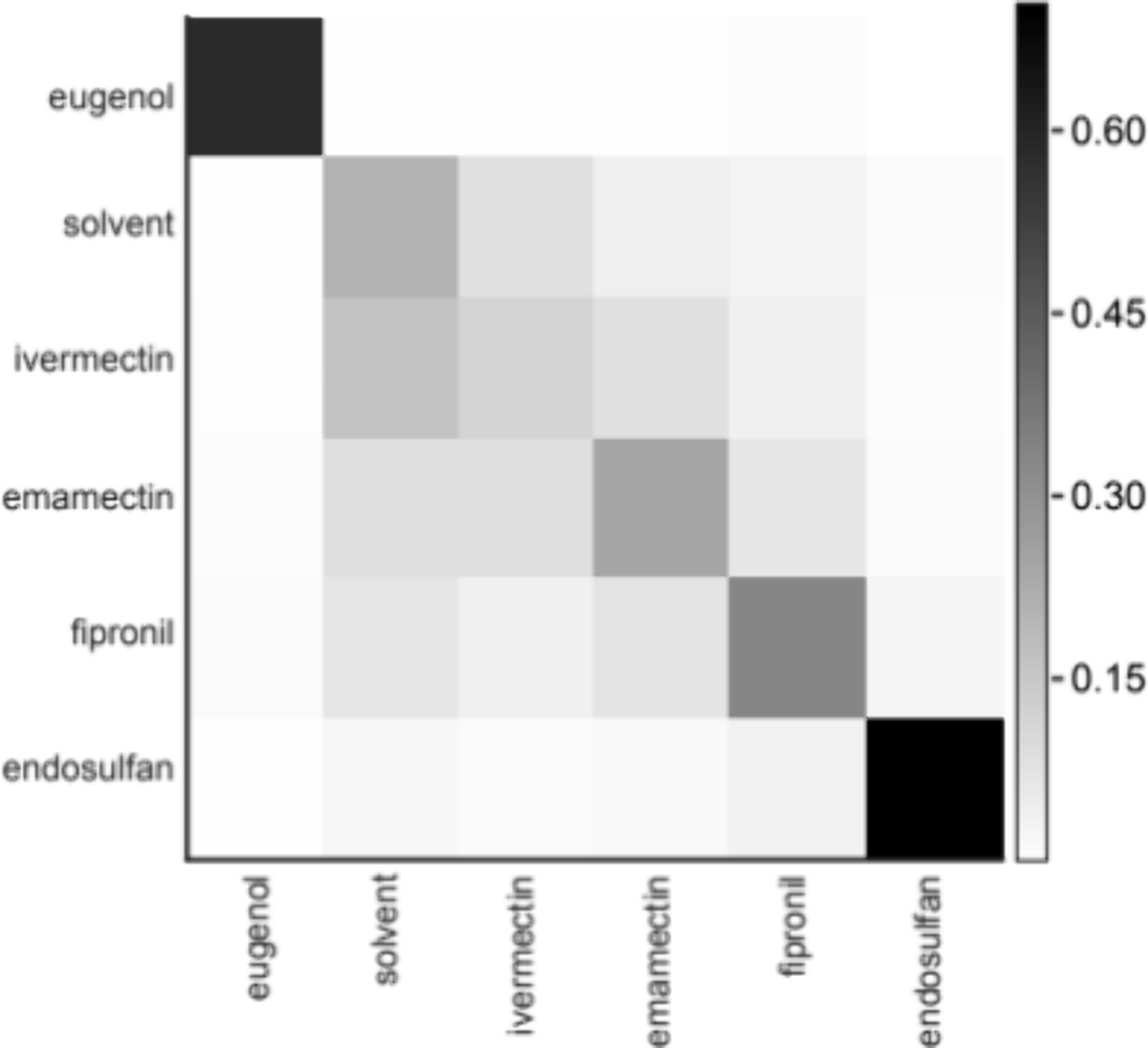
Random forest analysis of zebrafish behavioral response to chloride channel modulators. Confusion matrix from a multiclass classification model (random forests) performed on optimal concentrations of each treatment (*n* = 12–24 wells/condition).

**Figure 4:**
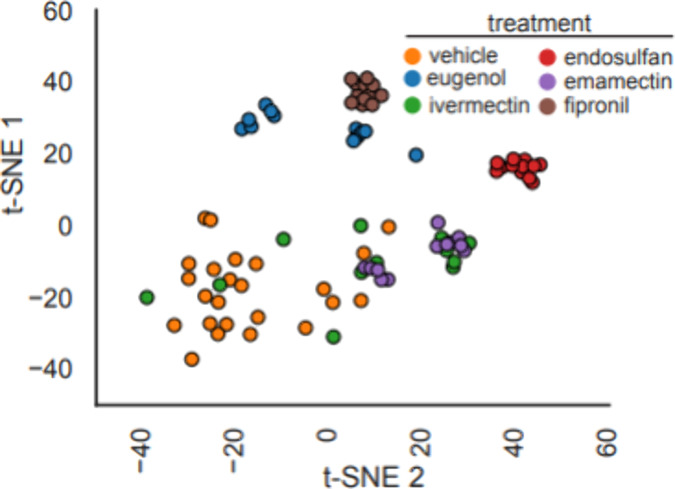
t-SNE analysis of zebrafish larvae behavior elicited by four mechanistically related insecticides. t-SNE projection of motion vectors looking at optimal concentrations for each treatment (*n* = 12–24 wells/condition).

**Figure 5:**
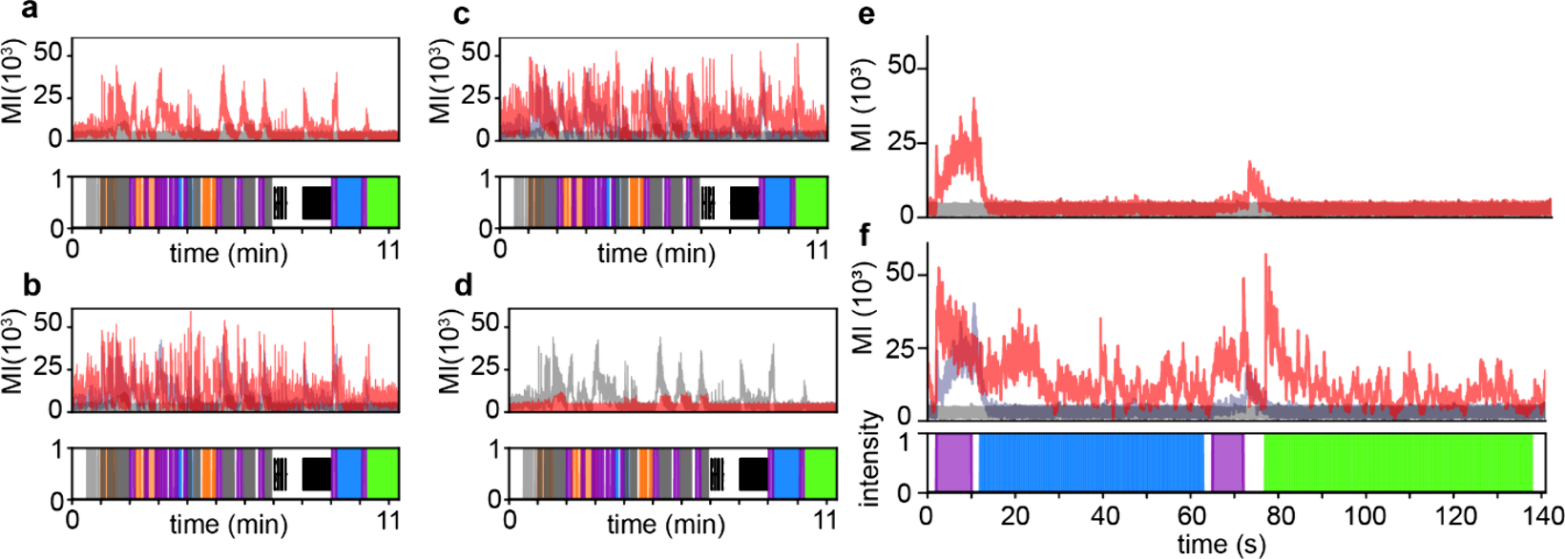
Motion indices (MI) of zebrafish phenotypic response. (a) Behavioral trace of an 11 min assay with vehicle treated animals in red, and lethal control in gray. (b–d) Increasing concentrations of endosulfan in red (195 nM in b, 390 nM in c, and 6,25 μM in d) with vehicle control plotted in gray. (e, f) Behavioral traces focusing in on the blue and green strobing assays comparing vehicle treated wells in red and lethal control in gray (e) and 195 nM endosulfan treated animals in red and vehicle in gray (f). Bottom plot is stimulus applied along the time axis. The shaded colors represent high-intensity LED light presentation, gray lines indicate solenoid tapping stimulus, and black indicates acoustic waveform application. Traces are an average motion index vector of *n* = 12 wells/condition.

Data visualizations for Figures 2–5 were generated using Python-based plotting libraries and Prism, then subsequently formatted for publication. Specifically, data were plotted using seaborn (version 0.10.1) and matplotlib (version 3.2) in a series of custom Python scripts designed to standardize aesthetic parameters (color schemes, line widths, and marker styles) across figures. Raw data files were read into Python (Version 2.8) and processed to the desired format, and visualized through reproducible code, ensuring transparency and consistency. The initial figure outputs were exported as vector graphics (PDF) and then arranged into multipanel layouts using Adobe Illustrator (2020). Within Illustrator, figure panels were aligned according to journal guidelines, and font choices and sizes were harmonized for clarity and visual coherence. Electrophysiology traces from Figure 8 were generated in Roboocyte II analysis software and annotated in Microsoft Paint. Figure 9 was generated using GraphPad Prism Software. Figure 10 used JMP Version 16, SAS Institute Inc., Cary NC, 1989–2023.

### Method for *Xenopus laevis* Oocyte-Based Assessment of GABA Block in *Drosophila* GABA Receptors

For cRNA synthesis the GABA receptor gene from *Drosophila melanogaster* (*rdl*) was cloned into pGH19. The construct was then linearized using XhoI. cRNA synthesis was performed using mMessage mMachine T7 Ultra kit (Ambion, Austin, TX) in duplicate. The cRNA reactions were then pooled and LiCl precipitated overnight at −20 °C. The pellets were resuspended to approximately 1 ng/nL in RNA storage solution (Ambion, Austin, TX). cRNAs were stored at −80 °C until use.

Defolliculated stage 5 oocytes were obtained from Ecocyte Bioscience (Austin, TX). Oocytes were injected with 2–10 nL of *D. melanogaster* poly A+ *rdl* receptor mRNA (1 ng/nL). Injected oocytes were housed individually in 96 well plates in ∼200 mL of ND-96 medium (96 mM NaCl, 2 mM KCl, 1.8 mM CaCl_2_, 1 mM MgCl_2_, and 5 mM HEPES, pH 7.6) supplemented with penicillin (10 units/mL) and streptomycin (10 mg/mL), and were incubated at 16 °C. After ≥24 h, injected oocytes were tested for GABA receptor expression using a Roboocyte II electrophysiology system (Multichannel Systems, Reutlingen, Germany).

For two electrode voltage-clamp recordings, ND-96 medium was replaced with Modified Barth’s Saline (MBS: 88 mM NaCl, 2.4 mM NaHCO_3_, 1 mM KCl, 0.41 mM CaCl_2_, 0.3 mM Ca(NO_3_)_2_, 0.82 mM MgSO_4_, and 15 mM HEPES, pH 7.6). Oocytes were voltage-clamped to −60 mV, and those with excessive leak currents (>−1000 nA) were not used for further study. Test compounds were first dissolved in DMSO and later diluted to the appropriate test concentration in MBS. DMSO levels never exceeded 0.1%. GABA was dissolved directly in MBS. During testing, oocytes were continuously perfused with MBS. Based on preliminary findings, 10 μM GABA was determined to yield currents that sustained their amplitude throughout extended application, allowing an assessment of current block by test compounds after establishment of sustained currents. Nonetheless, there was some desensitization of the GABA response using this paradigm. Therefore, GABA responses from nontreated GABAR-expressing oocytes were used to determine the mean amount of loss of response in the absence of potential antagonism, which was subtracted from each treated GABA/test compound treated oocyte (this is referred to as “rundown adjusted” on the Y axis of Figure 9). The amount of GABA-induced current block was quantified by the amplitude of the current immediately prior to the coapplication of the test compound (putative antagonist) expressed as a percentage of the current upon removal of the test compound. Percent block was then calculated by subtraction of the remaining current from 100%. Each compound was tested on 3–6 individual oocytes and the resulting data were expressed as the mean ± standard error of the mean.

### Method for Assessing Potency of Small Molecules Against *Diabrotica virgifera virgifera*

To determine the efficacy of compounds on Western corn rootworm (*Diabrotica virgifera virgifera*) larvae a diet-based assay was conducted. A nondiapausing strain reared at the Corteva Agriscience facility in Johnston, IA, USA was used for this test. Artificial diet was prepared according to manufacturer’s guideline for *D. virgifera virgifera* diet (Frontier, Newark, DE) with a few adjustments, including the addition of Formalin at 0.1% (v/v), 0.46% KOH (v/v), and triple antibiotic (Sigma-Aldrich, A5955) at 14% (v/v), and other proprietary improvements; and filled into Falcon Tissue Culture 96-Well Polystyrene Storage Microplates (Thermo Scientific, Waltham, MA). Technical material of the test substance was dissolved in a solution of acetone and deionized water (90:10 ratio); and 30 μL aliquots were pipetted onto the diet surface in the well. There were seven different concentrations which resulted in a rate range of 2–16 μg test compound/well. Additional wells were treated with only the diluent to obtain the natural background mortality in the test. For each treatment there were 12 replicates. Plates were air-dried, and three to five neonate larvae were added per well. Once infested, plates were heat sealed with clear vented plastic. The sealed plates were incubated at 25 °C, 60% relative humidity in the dark for 5 days. The mortality was scored based on the least affected individual for each well. No natural background mortality was observed on the diluent check in this test.

## Results

### Chloride Channel Insecticide Reference Set Analysis

To determine if insecticides known to act on insect chloride channel receptors can alter zebrafish larval behavior, emamectin, ivermectin, endosulfan, and fipronil were evaluated in full dose response (195.3 nM-600 μM) experiments in phenotypic zebrafish assays. These behavioral experiments were performed in seven-days post fertilization (dpf) zebrafish larvae using custom built hardware^12,13^ and a series of assays presenting acoustic and photic stimuli to the animals at different times to create a final behavioral battery lasting approximately 20 min. Initially lethal concentrations for each reference compound were determined in zebrafish larvae (Figure. 2). To accomplish this, we averaged the motion index (MI) values of the entire battery (consisting of over 38,000 data points) to obtain a single MI value. Average MI values of <1 was consistent with lethality. All compounds were lethal at concentrations ranging between 1 and 25 μM. Interestingly, endosulfan at concentrations between 195.3 nM-390.62 nM induced a dramatic increase in motor activity (Figure 2, red arrow, value = 310 average MI). This observation indicates a hyperactive/potentially seizurogenic state in the animal, consistent with previously reported inverse agonists of vertebrate chloride channels, such as PTZ.^30^

Random forest (RF) classifiers^31^ were then utilized to determine if these behaviors were distinct and predictable across multiple replicates. This analysis was completed in Python using the RF classifier included in the *sklearn* library. A confusion matrix (*X*-axis = known activity; *Y*-axis = predicted activity) was constructed using 500 trees and 4 parallel jobs (Figure. 3). In this analysis, eugenol was used as a control for lethality and DMSO solvent as a vehicle control. Eugenol is an inexpensive and widely available anesthetic and euthanasia agent commonly used with aquatic vertebrates, and it is reliable and predictable, making it a practical choice to avoid variation in the behavioral screen. Notably, endosulfan performed well in this analysis, indicating a unique and predictable phenotype for this compound when compared to other treatments.

Finally, t-distributed stochastic neighbor embedding (t-SNE) analysis on behavioral traces of the reference compounds with the controls at sublethal concentrations was performed to determine how behavioral changes induced by the commercial insecticides would cluster in a 2-dimensional space (Figure. 4). While vehicle-treated wells along with emamectin and ivermectin all appeared to occupy a similar space, unique clusters did appear with both endosulfan and fipronil. Fipronil occupied a similar space to the lethal eugenol-treated control while endosulfan was in a unique cluster with all instances of treatment tightly grouped. These results indicate that endosulfan has a unique and reproducible behavioral phenotype in zebrafish compared to other insecticides known to act on insect chloride channels.

### Endosulfan Induces General Hyperactivity and Greatly Increases Motor Activity in Response to Strobing Light Stimulus

To better understand the unique behavioral changes associated with sublethal doses of endosulfan, a motion index or MI was generated to represent zebrafish larvae activity across time in response to assay stimuli (Figure. 5). In these studies, lethal controls were used and resulted in a flat profile (gray trace, Figure. 5a), indicating complete loss of movement. In solvent-treated animals, motor activity was found to be dependent on the unique external stimuli applied (red trace, Figure. 5a). For example, in the presence of acoustic or purple light stimuli, increased motor activity was indicated by a dynamic profile (red trace, Figure. 5a). Interestingly, in zebrafish treated with sublethal doses of endosulfan, a dramatic increase in motor activity was seen across all assays, relative to solvent-treated animals (compare Figure. 5a to Figure. 5b, c). As the concentration increases, an overall loss of motor activity is measured, indicating toxicity (red trace, Figure. 5d, endosulfan 6.25 μM). Alternatively, in the presence of blue or green strobing light, the solvent-treated zebrafish were found to respond with complete loss of motor activity, as indicated by a flat red line (Figure 5e). Importantly, increased movement during the application of blue or green strobe stimuli was observed in the sublethal endosulfan-treated conditions (Figure. 5f, red trace). These results indicate that endosulfan causes an increase in both spontaneous motor activity as well as a specific increase in motion in response to a blue or green strobing light stimulus. An increase in motor activity is consistent with previously reported GABA inverse agonists such as PTZ and PTX.^19,30^

### Leveraging Unique Strobing Behavior to Identify Novel Endosulfan-Like Pharmacology

**Figure 6:**
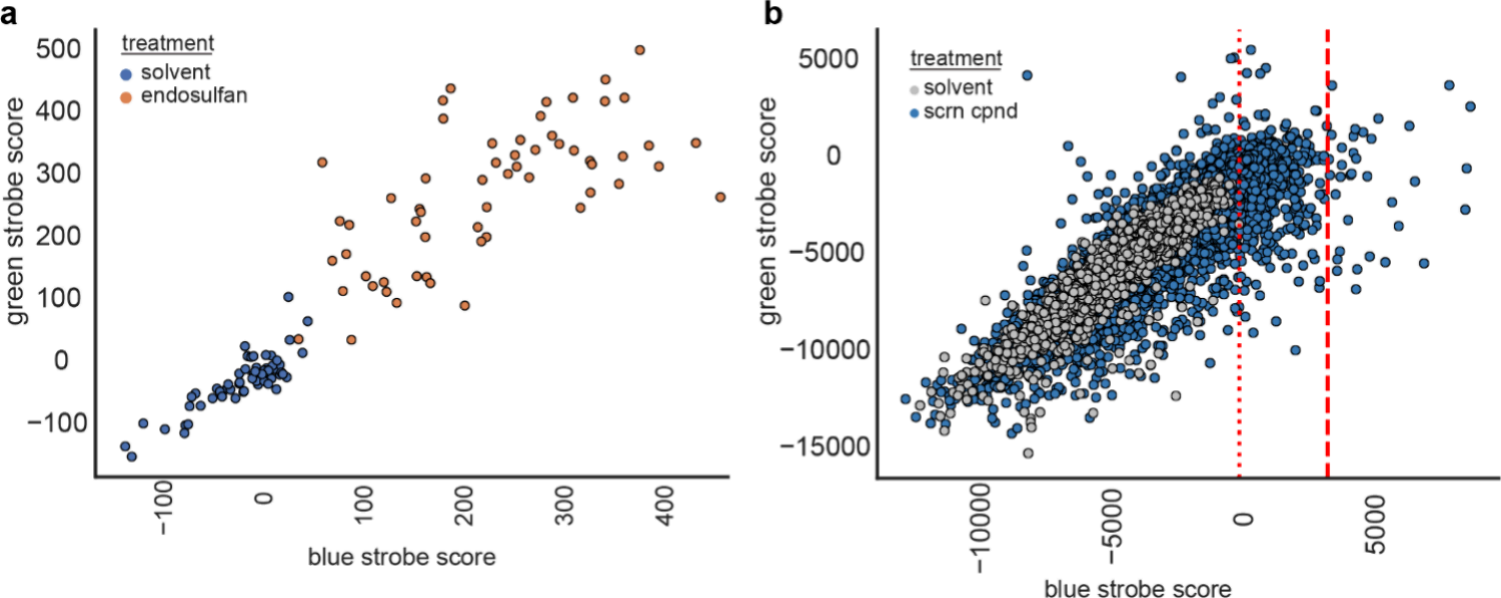
Compound screening based on scoring from blue and green strobing assays. (a) Scatterplot of multiple solvent control wells (blue) or endosulfan treated wells (orange) plotting the blue (*x*-axis) and green (*y*-axis) strobe scores (*n* = 58 wells/condition). (b) Scatterplot of full 9,512 compound screening library treated wells (blue) with 2336 solvent control wells (silver) plotting the blue (*x*-axis) and green (*y*-axis) strobe scores. The dotted red line indicates the location of the highest blue strobe scoring solvent treated well, the red dashed line indicates two standard deviations above the highest scoring solvent treated well.

Next, efforts were made to determine if this unique increase in motor activity in response to blue or green strobing light might be used to identify new compounds that phenocopy endosulfan in zebrafish. To answer this question, multiple replicate experiments were performed at different times to determine the reliability of a green vs blue strobe score to computationally separate behaviors of solvent-treated animals from those treated with endosulfan (Figure 6a). The average scores of the endosulfan-treated wells were significantly greater than that of the solvent control (274.1 ± 13.7 SEM vs −27.3 ±5.7 SEM) suggesting that a large-scale screen using this phenotype would have a low false positive and negative rate (Figure 6a, n = 58 wells). A library of 9512 structurally diverse compounds was then screened along with 2336 DMSO vehicle treated wells. The green strobe and blue strobe scores observed in this screening effort are summarized in Figure 6b. To interpret this data, the highest blue strobe score for a solvent treatment was identified (Figure. 6b, dotted line) and primary screening hits (35 total) were identified as exhibiting blue strobe scores at least 2 standard deviations (Figure. 6b, dashed line) above the highest solvent score.

**Figure 7:**
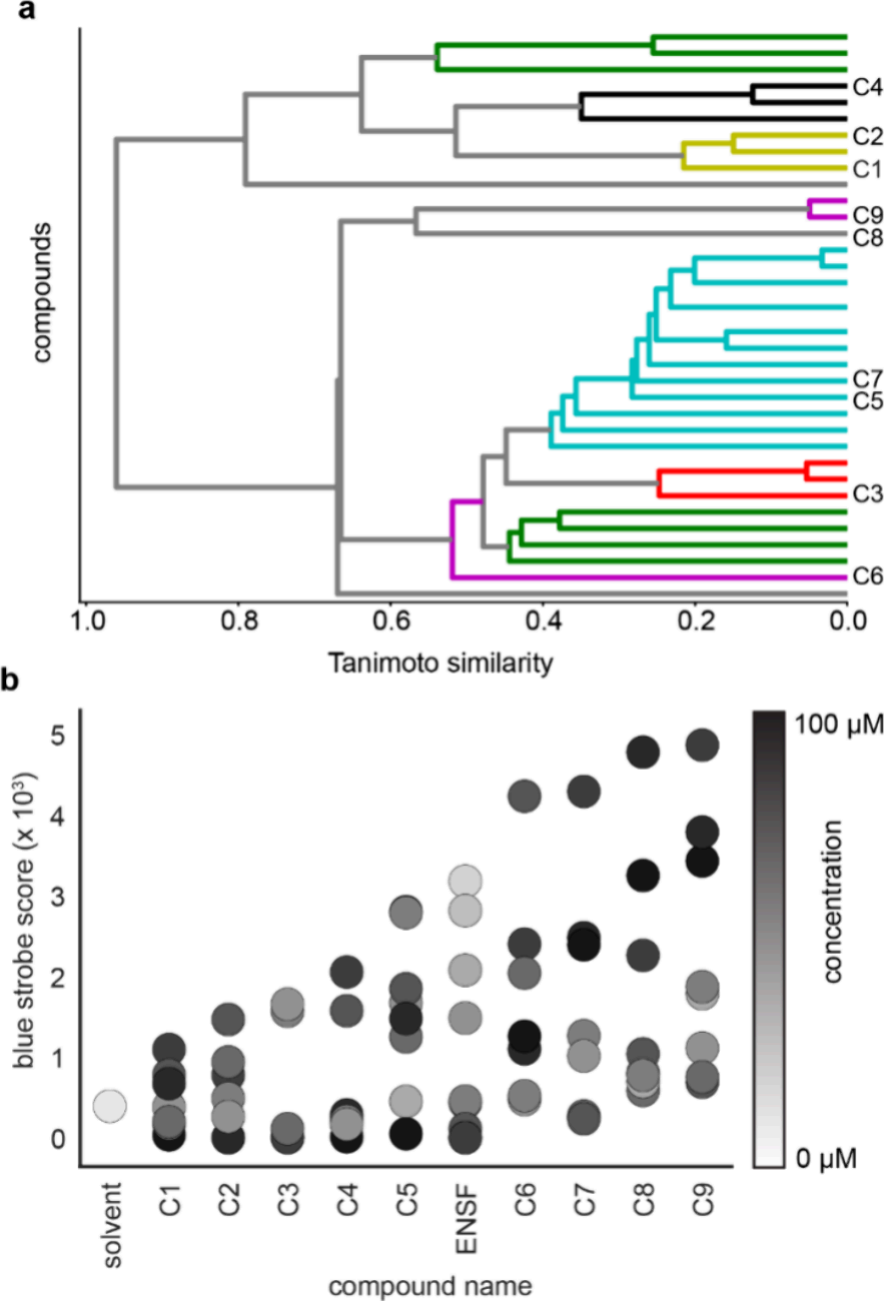
Structural analysis and retesting of screening hits. (a) Hierarchical clustering of hit compounds based on Tanimoto similarity coefficients (*x*-axis) and individual hit compounds (*y*-axis). Compounds selected for retesting experiments labeled with “c” number. (b) Retesting full dose response of 9 hit compounds (*x*-axis) compared to solvent and endosulfan (ENSF) controls at concentrations between 1.5 and 100 μM (color bar) and quantifying response to blue strobing light using a blue strobe score (*y*-axis, *n* = 6–12 wells/condition).

To assess structural diversity within these thirty-five compounds, clustering analysis was performed by calculating Tanimoto similarity coefficients, the results of which can be represented as a dendrogram (Figure. 7a). This analysis revealed 11 individual clusters. From these clusters, a representative set of nine molecules was selected for further characterization. These nine compounds were then included in full dose response behavioral experiments in the zebrafish larvae. Each compound was tested between a concentration of 1.0 μM-100 μM with 4 replicate wells and scored for their response to blue strobing light (Figure. 7b). All tested compounds had significantly higher strobe scores than vehicle-treated wells at several of the tested concentrations, indicating an endosulfan-like phenotype (refer to Figure 6a). Furthermore, four compounds (C6 - C9) were found to exhibit a stronger response than the endosulfan-treated positive controls (Figure. 7b). Together, all compounds retested caused a strobing phenotype stronger than that of the vehicle controls at one or more concentrations, with 44% of these primary hit compounds exhibiting a stronger phenotype than that of the positive control, indicating high reproducibility and potency in fish from the primary hits.

**Figure 8:**
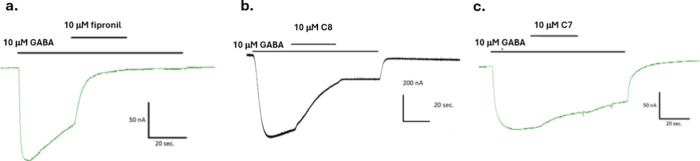
Insect chloride channel electrophysiology in response to fipronil and 2 hit compounds. Traces of inward currents of *Drosophila melanogaster* GABA receptors heterologously expressed in *Xenopus laevis* oocytes in response to GABA alone (10 μM), or GABA cotreated with fipronil control (a) or phenotypic hit compounds C8 and C7 (b, c, respectively).

With data indicating that these nine compounds induce phenotypes in zebrafish similar to the GABA receptor antagonist endosulfan, the ability of these molecules to also inhibit insect GABA receptor function was then assessed in electrophysiology experiments. For these studies, *Drosophila melanogaster* GABA receptors were heterologously expressed in *Xenopus laevis* oocytes. The two-electrode technique was employed to monitor GABA receptor function. As shown in Figure 8, sustained bath application of 10 μM GABA induced a sustained inward current that reversed only when GABA was removed from the bathing medium. However, coapplication of the known insect GABA receptor antagonist fipronil (10 μM) led to a rapid reversal (block) of the GABA-induced current (Figure 8a). Some, but not all, of the nine molecules that phenocopy endosulfan in the zebrafish assay were also demonstrated to produce substantial block of GABA-induced currents (e.g., compound **C8**, Figure 8b). Other phenocopy compounds were without effect against insect GABA receptor GABA-induced currents at the tested rates (e.g., compound **C7**, Figure 8c).

**Figure 9:**
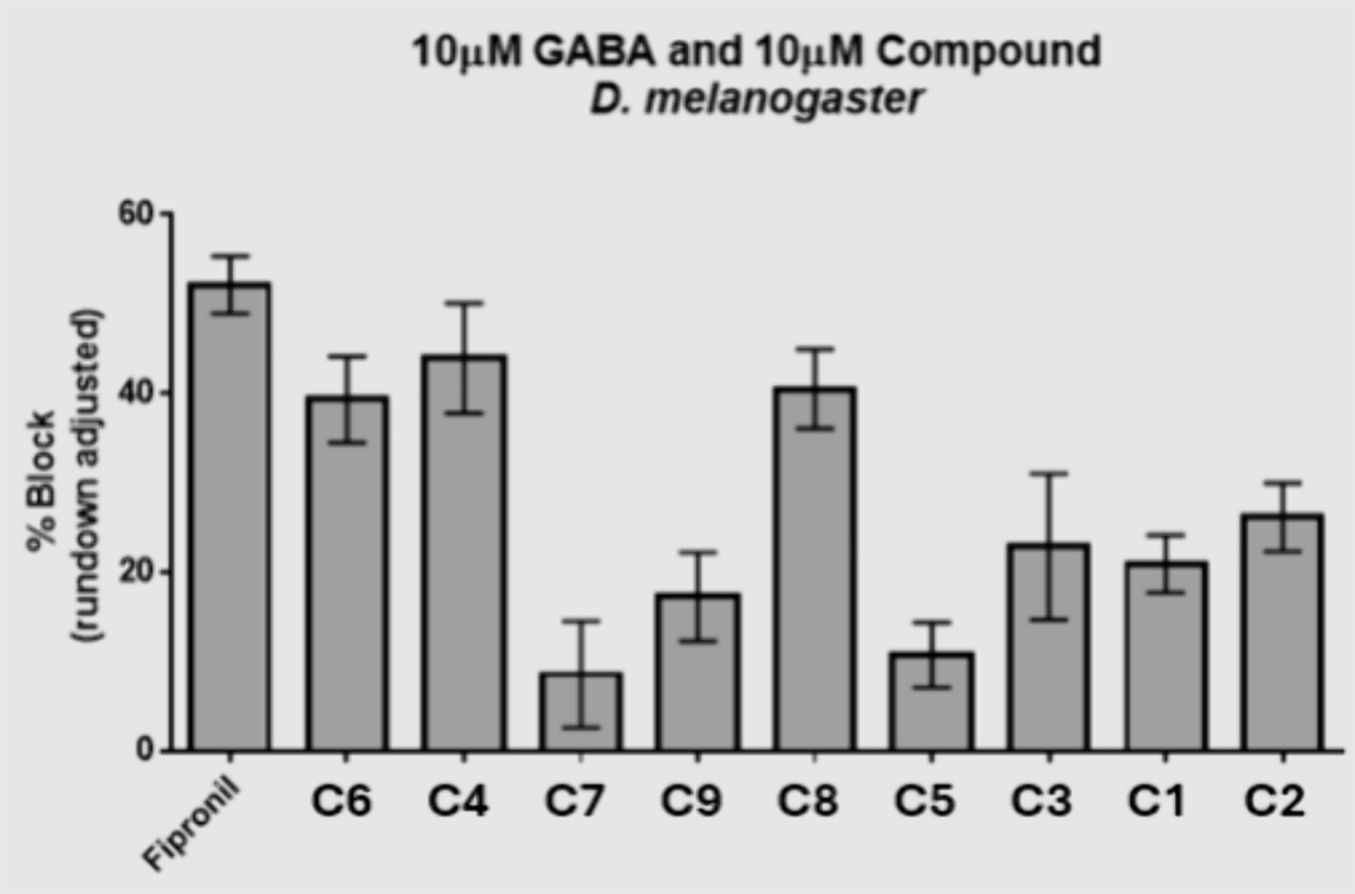
Assessment of GABA-inhibition by nine hit compounds. Average percent block of inward currents of*D. melanogaster*GABA receptors heterologously expressed in*Xenopus laevis*oocytes standardized to 10 μM GABA controls of phenotypic hit compounds C1–C9. Error bars represent ± standard error of the mean.

**Figure 10:**
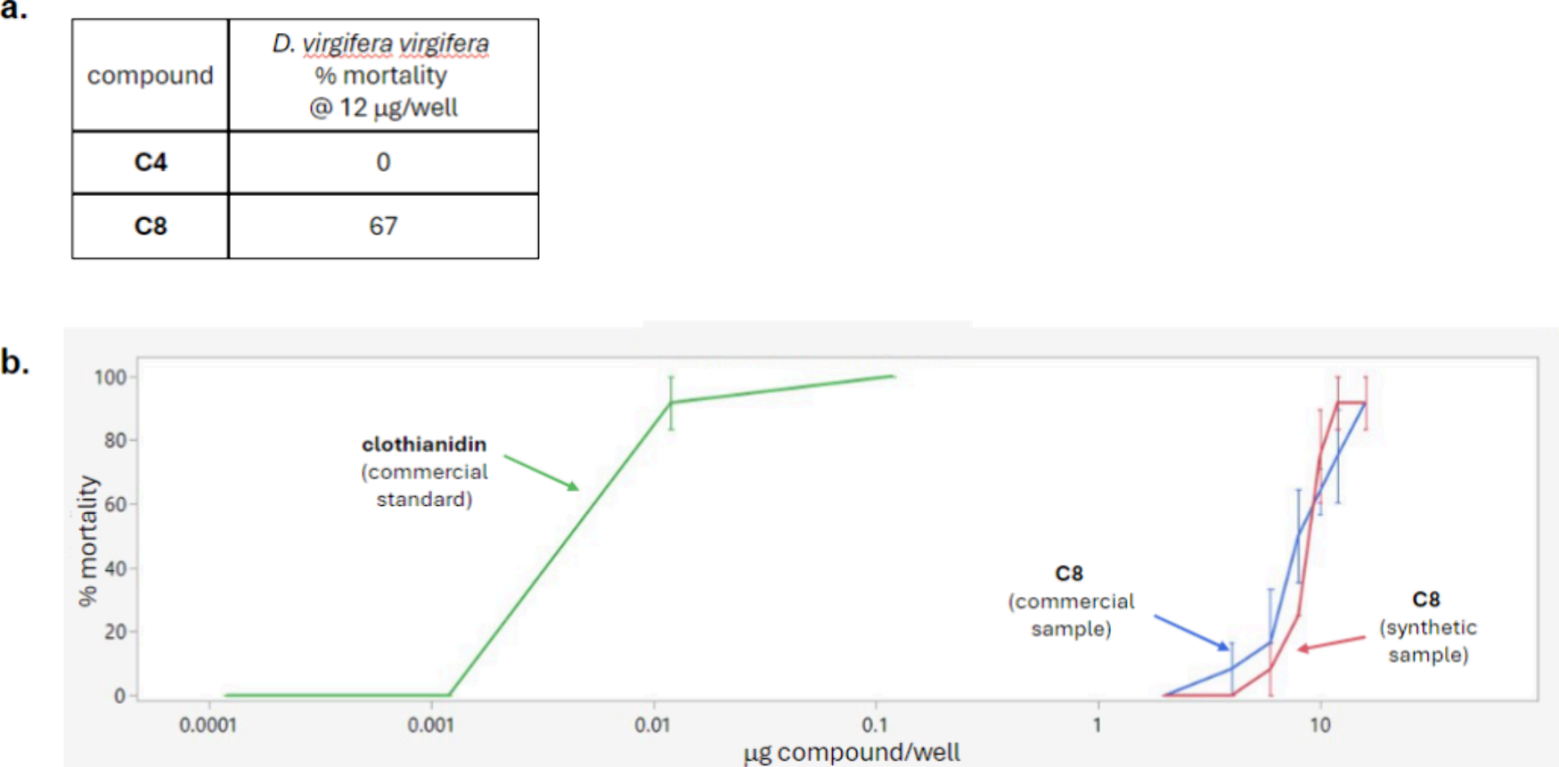
*In vivo* activity against the coleopteran pest Western corn rootworm (*Diabrotica virgifera virgifera*). (a) Single-rate mortality data. (b) Dose-response of compound C8 (commercial and synthetic sample with LC_50_ values of 8.61 and 8.94 μg/well, respectively) and the commercial standard clothianidin with an LC_50_ of 0.010 μg/well.

The ability of the nine endosulfan zebrafish phenocopy compounds to block insect GABA receptor GABA-induced currents is summarized in Figure 9. Interestingly, the three most active compounds (**C6, C4, C8**) produce robust GABA current block that appears to be similar to the known GABA receptor antagonist insecticide fipronil. Therefore, these three novel GABA receptor ligands may represent chemical starting points for the synthesis of more potent insect GABA receptor ligands, which could also lead to the discovery of novel insecticidal motifs. Based on the *in vitro* results above, efforts were made to assess activity in various *in vivo* insect assays. Of the compounds that showed *in vitro* GABA activity, we selected compounds **C4** and **C8** for these assays (**C6** was ultimately excluded due to equivocal results in the oocyte assay, data not shown). Gratifyingly, **C8** (commercial sample) was found to have *in vivo* activity against the coleopteran pest Western corn rootworm (*Diabrotica virgifera virgifera*) at a rate of 12 μg/well, while **C4** was inactive at this rate (Figure 10a). **C8** was retested at multiple rates to produce the dose response curve shown (Figure 10b, blue trace; calculated LC_50_ = 8.61 μg/well). This activity was confirmed on a separate sample of **C8** that was prepared through *de novo* synthesis (red trace; calculated LC_50_ = 8.94 μg/well). Though **C8** proved substantially weaker (>800X) than the commercial standard clothianidin (green trace; calculated LC_50_ = 0.010 μg/well) in this test, this result validates the hypothesis that novel compounds with activity against important insect pests can be identified using zebrafish with the appropriate stimulation. Therefore, it is conceivable that other molecules may be identified through a similar process.

## Discussion

Phenotypic screens have the capacity to identify new small molecules that achieve desired outcomes without requiring extensive mechanistic knowledge of the underlying biological process.^32^ Phenotypic screening of neuroactive compounds in a small scalable vertebrate animal offers a significant advantage over cellular models due to the presence of a central nervous system (CNS). Behavioral profiling in larval zebrafish has recently become recognized as an ideal system in which to perform CNS-based drug screens.^29,33^ Using this *in vivo* phenotype first platform, we identified a unique behavioral profile for endosulfan, a commercial insecticide known to act on insect chloride channels. We then leveraged this behavioral profile to identify new chemical matter with *in vivo* insecticidal activity. In this study, we focused on nine primary hit compounds with varying structures and efficacy in our assays. However, our screening campaign also revealed several other unexplored hits that could serve as potential leads. Further studies are needed to prioritize these compounds for potential development.

In addition to identifying new chemotypes for insecticide development, our profiling approach can potentially link behavioral perturbations to mechanisms of action. Our t-SNE analysis reveals that different insecticide treatments form distinct clusters, with GABA antagonist-related phenotypes, such as fipronil and endosulfan, clustering closely together. This proximity indicates that these data points share similar high-dimensional features which suggest shared characteristics. Similarly, ivermectin and emamectin, which are closely related, display overlapping groupings, suggesting they share similar behavior modifications. This data set thus demonstrates proof of concept. However, assembling larger reference sets of known pesticides and their effects on zebrafish larvae behavior could enhance the predictive power of our system and further establish the utility of this approach in discovering and understanding insecticides. Additionally, novel compounds identified from our large-scale, unbiased small molecule screen that phenocopy endosulfan have successfully recovered bona fide GABA antagonist ligands as demonstrated by our patch clamp studies. This underscores the potential of our approach to identify mechanistically related compounds. While this method may not be as precise as biophysical assessments, such as patch clamp studies at the biomolecular target, it serves as an excellent starting point for generating hypotheses on compound actions and provides a rapid first pass assessment that is easily scalable.

Identification of unique small molecule scaffolds for improved next generation pesticides has proven difficult, prompting a need for alternative methods of discovery for prototypic starting points. While the use of larval zebrafish toward the identification of insecticides may seem counterintuitive, this could be an innovative approach needed to accelerate insecticide discovery. Ideally, successful candidates will be specific to the pests of concern and not show off-target toxicity on other clades, such as vertebrates. However, after the initial hit scaffold is identified, structure–activity relationship studies can be implemented into the pipeline of insecticide development campaigns to optimize specificity to invertebrates while testing against unwanted larval zebrafish toxicity in parallel. This manuscript lays the groundwork for our approach, and we are committed to continuing this research by expanding upon this initial, proof-of-principle study in subsequent work. Overall, the implementation of zebrafish behavioral and toxicology assays in insecticide development is poised to be of great benefit to the field.

## Data Availability

All motion index time series data used in this study are available upon request.

## Notes

All code is available upon request.

## Abbreviations

CNS: central nervous system
dpf: days post fertilization
GABA: gamma-aminobutyric acid
GABAR: GABA receptor
SEM: standard error of the mean
RF: random forest
t-SNE: t-distributed stochastic neighbor embedding
MI: motion index
DMSO: dimethyl sulfoxide
MoA: mechanism of action
PTZ: pentylenetetrazole
PTX: picrotoxin
